# Post-Pandemic Maternity Care Planning for Vaccination: A Qualitative Study of the Experiences of Women, Partners, Health Care Professionals, and Policy Makers in the United Kingdom

**DOI:** 10.3390/vaccines12091042

**Published:** 2024-09-11

**Authors:** Tisha Dasgupta, Harriet Boulding, Abigail Easter, Tania Sutedja, Asma Khalil, Hiten D. Mistry, Gillian Horgan, Aricca D. Van Citters, Eugene C. Nelson, Peter von Dadelszen, Emma L. Duncan, The RESILIENT Study Group, Sergio A. Silverio, Laura A. Magee

**Affiliations:** 1 Department of Women & Children’s Health, School of Life Course & Population Sciences, King’s College London, London SE1 1UL, UK; 2 The Policy Institute, Faculty of Social Science & Public Policy, King’s College London, London WC2B 6LE, UK; 3 The RESILIENT Study Patient & Public Involvement & Engagement Advisory Group, UK; 4 Fetal Medicine Unit, St. George’s University Hospitals NHS Foundation Trust, London SW17 0QT, UK; 5 Fetal Medicine Unit, Liverpool Women’s NHS Foundation Trust, Liverpool L8 7SS, UK; 6 The Dartmouth Institute for Health Policy & Clinical Practice, Geisel School of Medicine, Dartmouth College, Hanover, NH 03756, USA; 7 Department of Twin Research & Genetic Epidemiology, School of Life Course & Population Sciences, King’s College London, London SE1 7EH, UK; 8 The RESILIENT Study Group, UK; 9 Department of Psychology, Institute of Population Health, University of Liverpool, Liverpool L69 7ZA, UK

**Keywords:** maternity care, COVID-19, vaccination, qualitative research, women, partners, healthcare professionals, policy makers, women’s health

## Abstract

Maternal vaccination during pregnancy, in general and against COVID-19 infection, offers protection to both mother and baby, but uptake remains suboptimal. This study aimed to explore the perceptions regarding COVID-19 vaccination in pregnancy, particularly for marginalised populations and those living with social or medical complexity. A total of 96 semi-structured in-depth interviews were conducted with 40 women, 15 partners, 21 HCPs, and 20 policy makers, across all four nations of the United Kingdom (UK), discussing their lived experience of utilising, delivering, or developing policy for COVID-19 vaccination in pregnancy during the pandemic. Three themes were derived: (1) historical and social context, (2) communication of information and guidance, and (3) appraisal and action. Together these captured the participants’ legacy of mistrust in drugs during pregnancy; prior positive experiences; concerns about missing information, conflicting information, or false information about COVID-19 vaccines; and confusing guidance for pregnant women. The final theme describes the participants’ behaviour and actions undertaken consequent to their experiences and the available information. The findings suggest efforts to improve COVID-19 vaccination in pregnancy may be best focused on personalised communication of information. A trusting relationship and prior positive experiences with other vaccines, both in and outside of pregnancy, positively influenced perceptions of COVID-19 vaccination.

## 1. Introduction

Given pregnancy-induced changes in immune modulation, pregnant women are more vulnerable to developing severe consequences from certain infectious diseases in pregnancy, including viral infections such as influenza and SARS-CoV-2 [1]. Maternal vaccination during pregnancy can mitigate these risks for the mother and provide some post-natal protection to their baby via transplacental transfer of maternal antibodies [1]. Although live attenuated vaccines have the potential for foetal infection and are therefore considered unsafe during pregnancy, considerable research supports the safety of inactivated vaccines [2,3]. In the UK, the National Health Service (NHS) recommends vaccination against pertussis at 16–32 weeks gestation for all pregnant women and against influenza for pregnancies that occur during winter (September–March). Nevertheless, vaccination during pregnancy remains suboptimal, with the annual vaccine coverage being 64.7% for those eligible for the prenatal pertussis vaccine in 2021–22 [3,4,5].

During the pandemic, rapid vaccine development and delivery reduced severe illness, hospitalisation, and mortality from SARS-CoV-2. The UK commenced public vaccination in December 2020 [6], tiered by age and vulnerability, initially with two doses and then a single booster. Initially vaccination was offered only to pregnant women if they were health care professionals or in an at-risk group, and it was then extended to all pregnant women by April 2021. As further evidence emerged regarding effectiveness (including the duration of protection), additional booster doses were recommended to vulnerable populations. By 2024, over 151 million doses of the COVID-19 vaccine had been administered in the UK [7].

By March 2023, approximately three quarters of pregnant women in the UK had received at least one (74%) or two (69%) doses of the COVID-19 vaccine by the time of their baby’s birth [8]. Nonetheless, 25% were completely unvaccinated by the time they gave birth, of whom only <1% were vaccinated postpartum [8]. In the UK, vaccination rates in pregnant women appeared slightly lower than for women of reproductive age in general [9]. In the latter group, reduced vaccination rates were associated with younger age, higher levels of deprivation, and some minority ancestry groups [6,9]. Negative impacts on COVID-19 vaccine uptake also included misinformation (e.g., via social media and the suggestion that the vaccine adversely affects menstrual cycling and/or fertility), perceived inadequate evidence about a relatively novel vaccine (e.g., about long-term outcomes), as well as discrepancies in information provided by different HCPs and inconsistent government guidance on the risks/benefits of COVID-19 vaccination in pregnancy [10,11,12].

We sought to explore the lived experiences and perceptions of women, partners, HCPs, and policy makers about the offer of the COVID-19 vaccine to pregnant women and birthing people, with specific emphasis on listening to individuals from marginalised communities or those who had experienced social and/or medical complexity.

## 2. Materials, Methods, and Procedure

This study was a component of the qualitative arm of the RESILIENT programme: “Post-pandemic planning for maternity care for local, regional, and national maternity systems across the four nations” [13].

Women, partners, HCPs, and policy makers were recruited for semi-structured in-depth interviews (conducted between May 2022 and February 2023), discussing their lived experience during the pandemic of using, accessing, delivering, or developing policy on COVID-19 vaccination (as applicable). They were also asked about their perceptions of a possible mandatory vaccination programme for maternity care staff in a future pandemic or health system shock. Thematic Framework Analysis [14] was used for analysis of the interview data, and it was stratified by participant type, ethnicity, geographic region, and social complexity. A qualitative study protocol for RESILIENT has been published with detailed methods including recruitment strategy, screening and consent process, procedures and dates of data collection, as well as data collection methods and analysis strategy [15].

A total of 96 interviews were completed with 40 women, 15 partners, 21 HCPs, and 20 policy makers. Table 1 below presents the demographic details of the participants.

The median participant age was 39 years (range 23–70 years). Overall, 65/96 (68%) participants identified as White or White British; 7 (7%) as Asian or Asian British; 12 (13%) as Black or Black British; 7 (7%) as mixed or multiple ethnicities; 4 (4%) as any other ethnicity; and 1 (1%) participant preferred to not disclose their ethnicity.

For women, partners, and HCPs, 38/76 (50%) interview participants used or were delivered maternity services in London, 27 (36%) in the rest of England, 4 (5%) in Wales, 4 (5%) in Scotland, and 3 (4%) in Northern Ireland.

For policy makers, their influence comprised national [n = 14, 70%], regional [n = 5, 25%], or local [n = 1, 5%] influence and reach.

Additionally, we collected information on the deprivation level, vaccination status, and COVID-19 high-risk status, gender, and sexual orientation. For policy makers, rather than their physical location in the country, we collected information on their work that created, implemented, or guided policy, including whether national, regional, or local in scope (see Table 1 for details).

## 3. Results

**Figure 1:**
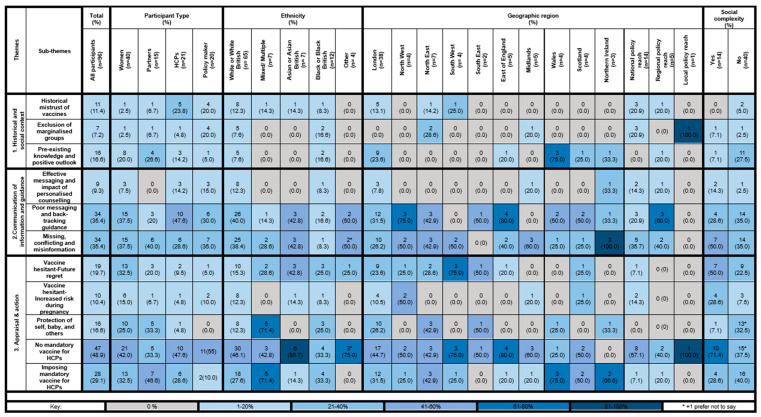
The participant’s lived experiences and perceptions of the offer of the COVID-19 vaccine in pregnancy.

We found three main themes and eleven sub-themes describing the lived experiences and perceptions of the offer of COVID-19 vaccination during pregnancy. Figure 1 presents the frequency of these themes and sub-themes, as mentioned by the participants, both overall and according to participant type, ethnicity, geographic region, and social complexity. For ease of interpretation, the frequency of the respondent reporting has been colour coded as grey (none), very light blue (1–20%), light blue (21–40%), blue (41–60%), dark blue (61–80%), and very dark blue (81–100%). Key supporting quotations for each sub-theme presented below.

The themes are structured in a chronological narrative, the first speaking to pre-pandemic, historical reasons for mistrust or positive perceptions towards vaccines; followed by participants’ experiences of the COVID-19 vaccination programme, information, and guidance during the pandemic; and, finally, the third theme describes the behaviours and actions taken in light of the first two themes, i.e., their historical context and experiences of the pandemic.

### 3.1. Historical and Social Context

The first theme describes the role of prominent historical events and social and cultural contextual factors that have shaped the way participants view vaccination in general, as well as COVID-19 vaccines and other drugs during pregnancy more specifically. This theme comprised three sub-themes: historical mistrust; the exclusion of marginalised groups; and pre-existing knowledge and positive outlook. Table 2 below presents key quotations for this theme.

Historical mistrust was reported infrequently (11% overall), and it was only by a minority of HCPs (24%) and policy makers (20%). This theme comprised data on the legacy of mistrust against Western medicine and care providers amongst certain minority groups attributed to historical medical malpractice and experimentation on people of colour. Only one woman and partner spoke to this issue. When analysed by ethnicity, the majority were White or White British (12%). Those who made relevant comments were mostly from London (13%) or were policy makers with national reach (21%).

Exclusion of marginalised groups was reported infrequently (7% overall). This includes individuals or groups who are less able to access and utilise basic services and opportunities in society, such as, but not limited to, belonging to ethnic minority groups, lower socioeconomic status, living in deprived areas, to those with learning or physical disabilities, and to those identifying as LGBTQIA+ [16]. Participants described the lack of targeted research, communication, or evidence for at-risk groups, including minority ethnic people and pregnant women. No Asian or other ethnicity, and only two Black participants, spoke to this point. By geographic area, most of the responses in this sub-theme were from those based in the northeast (29%) and policy makers with national reach (21%).

Pre-existing knowledge and positive outlook towards vaccines, even outside of pregnancy, was mentioned more commonly (17% overall), and it was mentioned by all participant types. The data in this theme describe how prior positive experiences and trust in HCPs, the health system, or government led participants to view the COVID-19 vaccine positively. Participants with Black/Black British ethnicity (17%), those from Wales (3 out of 4), policy makers with regional reach (33%), or those living without social complexity (27%) mentioned this sub-theme more often than those from other participant groups.

### 3.2. Communication of Information and Guidance

The theme which elicited the greatest number of responses focused on the communication of information and evidence of safety about COVID-19 vaccination, specifically for those planning pregnancy, those currently pregnant, or post-partum, along with comments about the need for guidance from the NHS and/or UK Government on when and who should be vaccinated. There were three sub-themes: effective messaging and impact of personalised counselling; poor messaging and back-tracking guidance; and missing, conflicting, and misinformation. Table 3 below presents key quotations for this theme.

Effective messaging and impact of personalised counselling was mentioned by a small number of participants (9%), i.e., only a few found that the communication was effective and the official guidance robust. This sub-theme summarises the participants’ positive views and experiences with the national guidance, in-depth counselling with HCP to combat missing information, and collaboration with community groups to effectively disseminate guidance. No participant of Asian/Asian British, Black/Black British, or other ethnicity resonated with this sentiment, and those who felt this way were predominantly residents of London (8%). Those living without social complexity (compared to those reporting living with it) were more likely to discuss this concept (14% vs. 3%).

Poor messaging and back-tracking guidance was raised by a large proportion of participants (35%), with comments highlighting that conflicting and/or confusing guidance contributed to vaccine hesitancy and overall lack of trust. Additionally, this sub-theme also collated data on national messaging, which was often different across the four nations of the UK, as being forceful and coercive, inducing mistrust, and how the lack of safety evidence should have been communicated more clearly. Approximately half of the women (38%), HCP (48%), and policy maker (35%) interviewees contributed data. Particularly high rates of comments were seen from Asian/Asian British ethnicity participants (43%) and policy makers with regional reach (3 out of 5). There was at least one individual from all regions of England (except the southwest and midlands) and high levels of respondents from Wales (2 of 4, 75%), Scotland (2 of 4, 50%), and Northern Ireland (1 of 3, 33%) who commented on this. Roughly equal numbers of respondents with (57%) and without (52%) social complexity reported on this.

Missing information, conflicting information, and misinformation was particularly well endorsed by participants (35% overall) who expressed the following: not having enough information available about COVID-19 vaccines, questions left unanswered by HCPs, a lack of personalised counselling, receipt of conflicting information from different sources, and receiving misinformation (particularly online). Participants also spoke to the lack of inclusion of pregnant women in drug trials and the, consequently, limited evidence that was available on the impact of the vaccine on an unborn foetus. This sub-theme was well endorsed across participant characteristics (with approximately 30% or more of all four participant types speaking to this topic). Policy makers, specifically those working at a local level, did not comment upon this matter.

### 3.3. Appraisal and Action

This third theme of Appraisal and Action describes the way participants behaved and the actions they took in light of the prior two themes, namely the historical and social context they lived in and the information and guidance they had from both official and non-official sources. This theme included their views on whether mandatory vaccination should be imposed for HCPs in a future pandemic or similar health system shock. The theme comprised five sub-themes: vaccine hesitant—future regret; vaccine hesitant—increased risk during pregnancy; protection of self, baby, and others; no mandatory vaccination for HCPs—autonomy; and mandatory vaccination for HCPs—duty of care. Table 4 below presents key quotations for this theme.

Future regret from being vaccinated was something a significant portion of the participants considered (20% overall), wherein they perceived the known impact of COVID-19 posed less of a risk during pregnancy than did the unknown (particularly long-term) risks of receiving vaccination during pregnancy. There was no pattern of response other than a greater endorsement of this sub-theme from the small number of individuals from the southwest of England (3 of 4, 75%).

Vaccine hesitant—increased risk during pregnancy was discussed by a minority of participants (10% overall). Vaccine avoidance during pregnancy was suggested as precautionary as pregnancy was perceived as a period of increased risk. Delaying vaccination was cited due to a fear of association with pregnancy loss or other complications, particularly when the participant had experienced a prior pregnancy complication. Many viewed the pregnancy period as too risky to receive a new vaccine, but were willing to do so after they had given birth to offer protection to their baby via antibody transfer in breastmilk. This was reported mostly by women (15%) rather than other participant groups, but there were no other particular patterns of response other than a small number of individuals from the northwest of England (2 of 4, 50%) who endorsed this theme. Those with social complexity reported on this sub-theme more often than those without (29% vs. 8%).

Protection of self, baby, and others was offered as a positive reason to receive vaccination by 17% participants overall. They felt being vaccinated posed less risk than COVID-19, particularly during pregnancy (and was seen to be of benefit to those most vulnerable to the virus). Policy makers did not contribute to this sub-theme.

No mandatory vaccine for HCPs was endorsed by almost half (49%). All the participants were asked to reflect on the proposed mandatory vaccination programme for maternity HCPs, with most being against it, citing HCPs’ rights to autonomy over their own bodies despite working in the healthcare sector, as well as the importance of education and counselling. Half or more of HCPs (48%) and policy makers (55%) reported on this sub-theme.

Imposing mandatory vaccine for HCPs, on the other hand, had fewer endorsements (29% overall), with some respondents mooting the acceptability of mandatory vaccination for maternity HCPs, citing that their duty of care to patients should take precedence. This was reported in particular by participants of mixed/multiple (71%) and those from Wales (3 of 4, 75%) and Northern Ireland (2 of 3, 66%).

## 4. Discussion

### 4.1. Summary of Findings

Our in-depth interviews with women, partners, HCPs, and policy makers illuminated three main themes encapsulating the participants’ perceptions regarding the offer of COVID-19 vaccination during pregnancy.

Most commonly, interviewees were concerned about inconsistent information and communication of information and guidance about vaccination, particularly missing information, conflicting information, and misinformation. Comments within the theme of the historical and social context of prior medical malpractice and discrimination were much less common and were mostly voiced by HCPs and policy makers rather than women and partners; moreover, fewer people from minority ethnic groups (when compared to White or White British participants) raised these concerns. Regarding actions taken in relation to COVID-19 vaccination, for some, hesitancy stemmed from a desire to avoid future regret of unforeseen complications from vaccination, or because pregnancy was perceived as a high-risk period warranting extra caution. However, for others, accepting vaccination was justified by prior positive experiences with other vaccines, trust in HCPs, the health system, government or medical research, and a desire to protect themselves, their baby, and other vulnerable or at-higher-risk members of society.

The interview schedule also explored a possible mandatory vaccination programme for HCPs and whether that should be implemented in a future pandemic. Our findings suggest that this practice was not highly endorsed, with precedence of body autonomy being taken over duty of care to patients, a rationale which was even cited by women and partners.

### 4.2. Interpretation

The findings from this study concord with other qualitative, quantitative, and mixed-methods studies in the UK [9,17,18] and globally [19,20], wherein vaccine hesitancy has been attributed to the following: a lack of clear guidelines from governments regarding recommendations for or against COVID-19 vaccination, confidence in the evidence of safety in pregnancy, trust in HCPs and the health care system in general, and attitudes towards other vaccinations offered routinely in pregnancy.

A systematic review of maternity care experiences during the COVID-19 pandemic [21], which was also part of the wider RESILIENT study programme of work, synthesised qualitative data from 27 studies in the UK and found similar results, noting five [4,12,17,18,22] of these 27 studies reported on vaccination. Our review showed that women who trusted vaccination in general appeared equally accepting of COVID-19 vaccination, perceived pregnancy as a high-risk period, and needed more information about the vaccine and its potential impact on their baby (before accepting the vaccine). Issues surrounding lack of trust in the government and pharmaceutical industry were raised, and they were fuelled by poor messaging and guidance [21].

A high proportion of data in this study contributed to the theme of missing information, conflicting information, and false information and guidelines about the COVID-19 vaccine for pregnant women. Participants reported on this theme across all participant groups, ethnicities, geographic regions, and social complexity. The UK Government’s guidelines for COVID-19 vaccination were particularly confusing for pregnant women. In December 2020, the UK Joint Committee on Vaccination and Immunisation (JCVI) initially released guidance stating that pregnant women should not be offered COVID-19 vaccination due to a lack of evidence of safety during pregnancy [23]. However, shortly thereafter, given the large body of evidence emanating from vaccine trials in the USA, guidance was changed to include all pregnant women [23]. This back and forth of guidance resulted in widespread confusion and contributed to vaccine hesitancy [17,18]. Additionally, vaccination recommendations were mandated by the JCVI rather than the Royal College of Obstetricians and Gynaecologists (RCOG) or the Royal College of Midwives (RCM), both of whom have greater expertise in pregnancy. Although messages were later distributed to local NHS Trusts and to service users via the official RCOG and RCM websites, there remained a discrepancy.

Previous research has shown trust to be a crucial motivator in vaccination acceptance [24]. A small number of participants in our study described being wary of vaccines and other drugs during pregnancy due to a shared collective memory of historical instances of medical mistreatment and poor practice such as the 1932–72 ‘Tuskegee Study’ for syphilis, in which affected people—mostly Black African Americans—were observed, but not treated for syphilis [25], and the 1950s–60s thalidomide tragedy, in which the thalidomide prescribed to pregnant women for morning sickness resulted in limb-reduction defects in their babies [26]. Another study linking vaccines with autism, published by Wakefield and colleagues in 1998 [27], caused widespread public concern, although it has since been proven otherwise. This history has left a legacy of mistrust among some participants, making them unlikely to participate in research or accept COVID-19 vaccines [25]. However, this issue was only raised by HCPs and policy makers, with a notable absence of comments from service users. Furthermore, the majority of responses were from White/White British ethnicity participants not from Black/Black British or other minority ethnicities. We must therefore question the origin of this discourse as to whether it is from the communities historically worse affected by adverse medical behaviours or whether it is a discourse derived from the residual guilt adopted by those not in minority ethnic groups and those responsible for new health care policy who are cognisant of the history. On the other hand, service users did reflect on current relationships with HCPs and trust placed in the government during the pandemic and its impact on the acceptance of national guidance and the COVID-19 vaccine.

Our finding that prior positive experiences and knowledge of vaccines, including routine vaccinations outside pregnancy, contributed to a positive view of COVID-19 vaccination is in-line with other research [4,12,21,28]. Women are less likely to accept vaccinations during pregnancy if they have received none outside pregnancy [29]. Furthermore, personalised counselling by an HCP, including comprehensive responses to questions, further addressed concerns. Our results echo other studies that show that a personalised and/or patient-centred communication model for health promotion is associated with increased adherence of health behaviours [30]. Research also shows that introducing vaccination as the social norm is largely more effective in combating hesitancy compared to directly dispelling misinformation [31]. This provides an excellent opportunity for public health and maternity care professionals to improve vaccination communication strategies, building on the precedence of other routine vaccinations in (and out of) pregnancy and creating a social norm by administering COVID-19 vaccination when and where other routine maternity care is provided. Currently, there are no existing recommendations for providing COVID-19 boosters to pregnant women as part of routine maternity care [32].

Finally, our study found evidence that imposing mandatory vaccination for HCPs, including maternity staff, in the event of another future pandemic would not be perceived favourably. To contextualise, the UK government proposed legislation in November 2021 making COVID-19 vaccination mandatory for all HCPs as a condition of employment [33]. While mandatory vaccines have been found to increase uptake in some situations such as if the mandate allowed individuals to travel or participate in recreational activities [34], concerns were raised that this would exacerbate the chronic workforce shortages in the NHS and cause thousands to lose their jobs should they refuse to comply with mandatory vaccination [35]. The government made a last-minute decision to reverse the legislation, bringing into question the governance and policymaking capabilities of the UK Parliament [36]. A large quantitative study of 3235 HCPs in the UK found only 1 in 6 HCPs favour mandatory vaccination over educating vaccine-hesitant HCPs [37]. The majority of participants in our study endorsed the belief that all staff should have autonomy over their own body and that this should take precedence over any duty of care towards patients. Rather, they too emphasised the importance of education and counselling of staff with questions or concerns.

### 4.3. Strengths, Limitations, and Future Research

To the best of our knowledge, this is the first in-depth qualitative exploration of the perceptions of the offer of COVID-19 vaccination during pregnancy from the perspective of multiple stakeholders. Although we were able to recruit women and partners from several groups who are seldom heard and/or find healthcare hard to access (by a careful recruitment strategy and working with local community groups), we found it difficult to recruit HCPs and policy makers in a similar manner, which could be a reflection of the people who hold these positions, particularly at a leadership level. Thus, our results for these groups may be less representative of views held by those from more marginalised sections of society. We would endorse future research in other countries with both similar free-at-point-of-use and pay-for-use health care systems. Likewise, post-pandemic exploration of public opinion remains important in relation to planning for future health system shocks and for the delivery of new vaccinations. Particular attention should be given to understanding the difference in implementation of guidance across the four nations of the UK. Future research should also explore the motivations of guilt or otherwise in those of the White/White British ethnicity echoing concerns over the legacy of the historical mistreatment of minority groups. We would urge future research to take a specific focus on controlling the spread of misinformation when it comes to COVID-19 and possible future pandemics [38]. The particular foci should be on understanding how false information is shared, accepted, and how it can be corrected [39]; how the social sciences can be employed to debunk health-related myths [40]; and how we can better equip ourselves to use social media to combat misinformation [41]. With this in mind, it is time for the social sciences and, particularly, the behavioural sciences, such as psychology, to take a more dominant role in risk- and health-communication, especially with regard to vaccines for novel illnesses such as the COVID-19 pandemic [42,43]. This will help to ensure our health systems have mastery over a range of proactive approaches and a management of vaccine roll-out and fielding queries [44] that is informed by high-quality science into the most cutting-edge behaviourally informed strategies [45], as well as ensuring we have a reach over vaccine education to marginalised groups, whilst also starting young so as to ensure children are provided with the correct information regarding vaccines [46].

## 5. Conclusions

Our findings suggest efforts to improve COVID-19 vaccination in pregnancy may be best focused on consistent and careful communication of information. Trust in HCPs, research, and the government plays a key role in service users’ acceptance of COVID-19 vaccination and to the vacillation and confusing messaging which has led to widespread confusion, anxiety, and vaccine hesitancy. There is an urgent need to include pregnant women in more drug trials in order to build a robust evidence base of the impact of drugs in pregnancy. On the other hand, prior positive experiences and perceptions of other routine vaccines are protective factors, making service users more likely to accept COVID-19 vaccination. Unified messaging, as had happened in other countries around the world with respect to COVID-19 vaccines in pregnancy (e.g., Canada), will help with a better public health response in future health system shocks in the UK. Finally, our study indicates that, in a future pandemic, mandatory vaccination programmes for healthcare staff are unlikely to be accepted, and efforts should rather be focused on education and health promotion.

## Figures and Tables

**Table 1:** Demographics of the interview participants.

	Women (N = 40)	Partners (N = 15)	HCPs (N = 21)	Policy Makers (N = 20)	Total (N = 96)
**Age at Interview**					
18–25	2	0	0	0	2
26–30	5	6	1	0	12
31–40	28	7	5	1	41
41–50	5	2	4	5	16
51–60	0	0	8	8	16
61–70	0	0	2	3	5
Prefer not to say	0	0	1	3	4
**Gender**					
Female	40	14	15	15	84
Male	0	1	5	5	11
Non-binary	0	0	1	0	1
**Sexual Orientation**					
Heterosexual	36	11	18	15	80
Bisexual	3	3	3	0	9
Lesbian	1	0	0	1	2
Gay	0	1	0	1	1
Prefer not to say	0	0	0	4	4
**Ethnicity**					
White/White British	24	7	17	17	65
English/Welsh/Scottish/Northern Irish/British	19	5	12	13	49
Irish	1	0	1	1	3
Any other White background	4	2	4	3	13
Asian/Asian British	6	0	0	1	7
Any other Asian background	0	0	0	1	1
Bangladeshi	2	0	0	0	2
Chinese	1	0	0	0	1
Indian	3	0	0	0	3
Black/Black British	6	4	1	1	12
African	1	2	0	0	3
Any other Black/African/Caribbean background	2	1	1	0	4
Caribbean	3	1	0	1	5
Mixed/Multiple ethnicity	2	4	1	0	7
Any other Mixed/Multiple ethnic background	0	0	1	0	1
White and Asian	1	1	0	0	2
White and Black African	0	1	0	0	1
White and Black Caribbean	1	2	0	0	3
Other ethnic group	2	0	2	0	4
Any other ethnic group	1	0	1	0	2
Arab	1	0	1	0	2
Prefer not to say	0	0	0	1	1
**Geographic Region**					
London	18	8	12	n/a	38
East of England	2	1	2	n/a	5
Midlands	3	0	2	n/a	5
North East	4	2	1	n/a	7
North West	3	1	0	n/a	4
South East	2	0	0	n/a	2
South West	2	1	1	n/a	4
Wales	3	0	1	n/a	4
Scotland	2	1	1	n/a	4
Northern Ireland	1	1	1	n/a	3
National reach (policy makers) *	n/a	n/a	n/a	14	14
Regional reach (policy makers) *	n/a	n/a	n/a	5	5
Local reach (policy makers) *	n/a	n/a	n/a	1	1
**IMD**					
1 (most deprived)	3	0	0	0	3
2	3	1	0	0	4
3	2	0	1	3	6
4	4	1	1	1	7
5	5	5	3	1	14
6	5	0	2	1	8
7	4	4	4	2	14
8	6	3	3	0	12
9	5	0	0	4	9
10 (least deprived)	3	0	2	1	6
Prefer not to say	0	1	5	7	13
Social Complexity (only Women and Partners) **					
Yes	14	0	n/a	n/a	14
No	25	15	n/a	n/a	40
Prefer not to say	1	0	n/a	n/a	1
**Personal/Household High-Risk Status**					
Yes	8	4	6	5	23
No	32	11	15	13	71
Prefer not to say	0	0	1	2	2
**Vaccination Status**					
Yes—full dose including booster	33	12	20	19	84
Yes—no booster	2	1	0	0	3
None	4	2	1	0	7
Prefer not to say	1	0	0	1	2

* For policy makers, data were collected on the geographic scope of their work, rather than physical residence. ** Social complexity was self-identified by participants and included the following: lack of social support, mental health problems, or belonging to a minority group relating to sexual orientation or gender identity.

**Table 2:** Interview key quotations: Theme 1—Historical and social context.

Themes	Sub-Themes	Quotations
1. Historical and social context	Historical mistrust of vaccines	Interestingly, very common among Afro-Caribbean women, in particular, that they feel that it’s not something for them, because of that problem. And of course, that comes in with the history of vaccination usage over the last century, of how black people were used, for example, as guinea pigs, and I think that inheritance of information, makes people a little bit sceptic and fearful of using the vaccine.—H007
	Exclusion of marginalised groups	We take a very cautious approach to vaccines and pregnancy, which is right. Everybody remembers the thalidomide scandal, and the last thing we want to do is offer vaccines which are safe in the general population to pregnant women where it turns out not to be safe. What this does though is it leads to a bit of caution in terms of clinical trials and at the end of the day, you do a trial, you exclude pregnant women and you don’t know whether the vaccine is safe in pregnancy. Over time, that rectifies itself because some women who didn’t know they were pregnant when they had the vaccine turn out to be pregnant, and you build up the evidence base that way. In this case, there was such an enormous time pressure to develop a vaccine and roll it out, so we ended up being in the position where we had to roll out the vaccine without knowing it was safe for pregnant women, so you had to tell pregnant women “We’re not offering you the vaccine at the moment.—M012
	Pre-existing knowledge and positive outlook	But also, I think the thing that was missing is that a lot of people were saying, “Well, it’s untested,” and that wasn’t actually true. There’s a lot of that technology that we are already aware of, and we can apply it to lots of different diseases. So I didn’t have those sorts of concerns that other people had. I did find the [Vaccine] blood clots quite alarming, like everybody. And when I was able to get a vaccine, I had the [Vaccine] one. And I was very relieved about that because I am at a much higher risk of blood clots because of the various conditions I had during pregnancy.—W021

Key: W = women, P = partner, H = HCP, and M = policy maker.

**Table 3:** Interview key quotations: Theme 2—Communication of information and guidance.

Themes	Sub-Themes	Quotations
2. Communication of information and guidance	Effective messaging and impact of personalised counselling	No, I was just going to say, I think the messaging was sound. When I got the messaging throughout my pregnancy about vaccinations I needed, that came from the midwives, from the health care providers, it wasn’t something that I looked for via the government. I was just told by the healthcare professionals ‘please book yourself in for flu, book yourself in whooping cough’. And whatever else there was. But yes, I had the standard recommendations, yes.—W031
	Poor messaging and back-tracking guidance	So I think what would have been really helpful at the start of the pandemic is for there to be a really open transparent phase but also to do with vaccination, to be like, ‘This is what we know at the moment and these are the current signs and symptoms’ or ‘This is the current guidance on vaccination. As the pandemic evolves, this advice may change’ and I feel like that would manage the public’s expectation way more for then three months down the line when we are like, ‘Oh no, pregnant women now can have the vaccine’, people wouldn’t be like, ‘Oh my gosh, it’s all changing, oh my gosh. What’s all this conflicting advice? No one knows what they are doing’. It’s like, ‘No, this is a natural… This is how pandemics naturally evolve in terms of information available and how it’s then translated into the public domain’. But because we didn’t express that uncertainty at the beginning, because you don’t want people to be anxious and you want people to believe public messaging and trust it—H013
	Missing, conflicting, and misinformation	It’s too experimental for my liking. There’s lots of things going wrong with people, and I know that they say that it’s nothing to do with it, but… My mum for example has ended up with fluid round her heart, and they’ve told her it’s because of the vaccines, and she can’t have any more. My husband ended up in hospital with heart problems after having his second vaccine. They didn’t say it was the vaccine, but it doesn’t take a genius to work it out. He hasn’t had any more. And we know a couple of people round here that after they’ve had their injections, they’ve ended up with brain bleeds.—W035

Key: W = women, P = partner, H = HCP, and M = policy maker.

**Table 4:** Interview key quotations: Theme 3—Appraisal and action.

Themes	Sub-Themes	Quotations
3. Appraisal and action	Vaccine hesitant – Future regret	We decided, as a couple, as a family, that we would wait. We were not going to jump straight into getting basically anything stuck into our arm that hadn’t been clearly tested for some time. We were not going to be the initial guinea pigs. We saw some data that was coming out about complications, side effects, even death. And I thought, “Do you know what? I’m not prepared to put any of us in that situation.” So, now that we’re so far in and there’s a lot more data and we’ve got a really good idea of complications, and even, you know, fatal complications. I think that arguably it makes sense.—P014
	Vaccine hesitant—increased risk during pregnancy	And we weren’t comfortable in having the vaccine while I was pregnant, as we didn’t feel there was enough research on pregnant women and the side effects that it can have, and I’d already had Covid twice, so I didn’t think… I had enough resistance, I didn’t really have any side effects from Covid, I was really quite well during it. But the consultant sort of pushed me into getting the second vaccine… So I went and had the vaccine, and then the day later, I felt no movements—the day after my vaccine. And when I went into the hospital, they just couldn’t find the heartbeat… I just felt that the vaccine hadn’t been out long enough for me to take it especially when I’d had it twice and I didn’t feel… if I’d had really bad side effects, and that could have affected my pregnancy, I would have had the vaccine, no problem, if it was more risky for me to catch it.—W010
	Protection of self, babies, and others	I’m very pro-vaccine so personally I would totally encourage it if it protects you and protects your baby. I think we’re so lucky to be in a developed country where we’ve got access to vaccines. I do hear the argument of, it’s my body and it should be my choice. I totally hear that. I suppose, I just think for the greater good, not just for that person or for their baby but for everyone, the more people who are vaccinated, the less hosts there are for the virus to infect and create a new variant. So vaccines have eliminated illnesses in the past and so yes, I’m very pro-vaccine and I’d encourage any woman who is pregnant or thinking about getting pregnant, I would definitely encourage them to take it up, for sure, yes. If they feel it’s the right thing, yes.—W031
	No mandatory vaccine for HCPs – autonomy	I don’t think it’s right to force healthcare workers, even in maternity environments and high-risk environments, to be vaccinated. Yes, they have a right to protect the patients that they look after. You would hope it would be the minority of healthcare professionals that wouldn’t be vaccinated because I think most people working in healthcare altruistically do want the best for people and want to do the best for patients and the people they look after and there’s a sort of caring element to health care… I think to force it, I think it just felt wrong to me that it’s mandatory for you to have vaccination and I was really pleased actually when the government u-turned on that aspect of things, because we were getting a lot of people saying that it wasn’t fair, vaccination is a choice, and I think it is a choice and my view is that you do have to give healthcare workers a choice. If they don’t want to be vaccinated then I think that they have the right not to be vaccinated. But then they also have a need to protect their patients so they need to be doing regular Covid tests, they need to not be exposing patients to Covid. But to go as far as to say that they all have to be vaccinated I thought was too far.—H009
	Imposing mandatory vaccines for HCPs – duty of care	I feel like the need to not put your patients at risk takes priority over… I feel like the argument against it is that people have a right to refuse to take a vaccine. And I absolutely agree that that’s the case, that people should have a choice about whether to take it or not. But then, to say that people’s right to exercise that choice means that they should continue to be able to work in an environment where that might put other people at risk, is a kind of secondary question and a separate question. So to the same extent that—there’s not really a good analogy for it really, is there? But basically I think that it’s fine if someone doesn’t want to take the vaccine, but then they also shouldn’t be working in an environment where that would put people at much more risk. And I don’t know then that means that it’s the responsibility of their employer to move them to a role where they wouldn’t then be exposed to patients. That might be a kind of compromise. But I think definitely frontline staff should be vaccinated.—P010

Key: W = women, P = partner, H = HCP, and M = policy maker.

## Data Availability

The data presented in this study are available on request from the corresponding authors due to the sensitivity of the dataset.

## Appendix A

The RESILIENT Study Group consists of: *Chief Investigator:* Laura A. Magee (King’s College London); and *Co-Investigators:* Prof. Debra E. Bick (The University of Warwick), Dr. Harriet Boulding (King’s College London), Dr. Kathryn Dalrymple (King’s College London), Ms. Tisha Dasgupta (King’s College London), Prof. Emma L. Duncan (King’s College London), Dr. Abigail Easter (King’s College London), Prof. Julia Fox-Rushby (King’s College London), Miss. Gillian Horgan (King’s College London), Prof. Asma Khalil (St. George’s University Hospitals NHS Foundation Trust & Liverpool Women’s NHS Foundation Trust), Ms. Alice McGreevy (King’s College London), Dr. Hiten D. Mistry (King’s College London), Prof. Eugene C. Nelson (Dartmouth College), Prof. Lucilla Poston (King’s College London), Mr. Paul Seed (King’s College London), Sergio A. Silverio (King’s College London & University of Liverpool), Dr. Marina Soley-Bori (King’s College London), Dr. Florence Tydeman (King’s College London), Ms. Aricca D. Van Citters (Dartmouth College), Dr. Sara White (King’s College London), Prof. Ingrid Wolfe (King’s College London), Prof. Yangzhong Wang (King’s College London), & Prof. Peter von Dadelszen (King’s College London).
